# Relationship between the Apical Preparation Diameter and the Apical Seal: An In Vitro Study

**DOI:** 10.1155/2018/2327854

**Published:** 2018-01-10

**Authors:** Kaoutar Laslami, Sara Dhoum, Amine El Harchi, Iman Benkiran

**Affiliations:** ^1^Department of Conservative Dentistry and Endodontics, School of Dentistry of Casablanca, Casablanca, Morocco; ^2^Private Practice, Casablanca, Morocco

## Abstract

**Objectives:**

The aim of the study is to define the relationship between the apical preparation diameter and the apical sealing ability to highlight the importance of the preservation of the diameter and the original position of the apical foramen.

**Materials and Methods:**

50 extracted maxillary incisors were randomly allocated into three groups of 15 teeth each (n = 15) according to the apical preparation size: Group 1: finishing file F1 corresponding to size 20 reached the working length (ProTaper Universal system Dentsply®); Group 2: prepared up to size 30 corresponding to finishing file F30; Group 3: prepared up to size 50 corresponding to finishing file F5. Five teeth were assigned to positive and negative control groups. After the filling of the root canals, the teeth were isolated and immersed in a dye solution, then cut longitudinally, photographed, and the dye penetration were calculated using a computer software.

**Results:**

Comparison of the three different apical preparation sizes showed no statistically significant differences regarding the apical microleakage.

**Conclusion:**

The most important value of the dye penetration was observed in the group with the largest apical diameter.

## 1. Introduction

The root canal filling achieves three significant objectives:

- Elimination of residual bacteria from the root canal system
- Prevent the entry of fluid from the periapical tissues
- Prevent coronal microleakage [1, 2].

Three-dimensional and hermetical seal increases the tooth longevity [3]. However, the root canal filling cannot compensate a lack of canal preparation. Thus, it is important to understand that preparation and root canal filling complement one another [4].

It has been shown that 63% of the endodontic treatment failures are related to apical percolation due to an insufficient sealing [5]. It thus appears that the shaping and disinfection of the apical third takes a paramount importance in the success of the endodontic treatment [6].

Two questions arise here: Which apical limit and which apical diameter size should we choose?

For the apical limit, the current recommendations require ideally that instrumentation and obturation should not exceed the root canal space [6, 7]. The shaping of the last apical millimeters should respect mechanical and biological objectives: conicity, respect of the apical diameter and the original position of the foramen are important for the adjustment of the master cone in the apical third [3].

On the other hand, if the main objective of endodontic therapy is to create a favorable environment in the apical healing process, all instrumentation beyond the foramen causes damages to the periapical tissues due to extrusion of the debris and the irrigation solutions and impairs the adequate seal of the apical foramen [7, 8].

As for the apical preparation diameter, one of the main mechanical imperatives in the apical third preparation is to preserve the apical foramen in its initial position and in its narrowest diameter in order to avoid any complication such as tearing, zipping, or transport of the foramen [9, 10].

However, we should not forget that bacteria penetrate deeply in the dentinal tubules, especially when the teeth are infected, and that some authors support the necessity of an apical enlargement in order to reduce as much as we can the bacterial growth in the apical third [11–13].

All things considered, the diameter preparation of the foramen is a very controversial topic in endodontics.

The aim of our study is to compare the apical leakage after the preparation following different apical diameters.

## 2. Materials and Methods

Freshly extracted 50 central incisors were selected and stored in a physiological serum. All the teeth were free of cracks and had single root canals and mature apices.

The teeth were randomly allocated into 3 experimental groups of 15 teeth each and 2 control groups:

  Group 1: Canals were prepared up to size 20 corresponding to finishing file F1 of the ProTaper system (Dentsply).
  Group 2: Canals were prepared up to size 30 corresponding to F3.
  Group 3: Canals were prepared up to size 50 corresponding to F5.
  Group 4: The positive control group contained 3 roots that were not filled and covered with nail polish except for their apical 2 mm.
  Group 5: The negative control group included 2 roots that were not filled, and the entire root surface was coated with nail polish, and canal orifices were sealed with cyanoacrylate glue.

### 2.1. Root Canal Preparation

Endodontic access cavities were realized using high-speed burs under water spray, and all root canals were instrumented by the same operator. Patency of each canal was established by gently passing a 08 k-file, and the working length was determined using a 10 k-file (Dentsply Maillefer) into the canal until it becomes visible through the apical foramen, then subtracting 1 mm.

The canals were instrumented using the crown-down technique with rotary ProTaper nickel-titanium files (Dentsply Maillefer) up to the apical size of the F1 finishing file, and F3 or F5 in order to increase the apical diameter depending on the desired final apical diameter for each group. The canals were irrigated between files with 2 ml of 5.25% sodium hypochlorite (NaOCl). Following instrumentation, the smear layer was removed with 17% EDTA, followed by irrigation with NaOCl at 5.25%. Then, they were dried with sterile paper points. The master-cone adjustment is done thanks to the tug-back test. Then, the root canal filling was performed using the cold lateral condensation technique.

### 2.2. Sectioning and Image Analysis

To ensure that all specimens were of the same length, they were resected 17 mm from the apex using a diamond disk. Two layers of nail polish were applied to the root surface, except for the apical 2 mm that remained exposed to the dye solution. The 2 teeth used as negative controls had the entire root surface sealed.

Specimens were then immersed in 1% methylene blue for 48 hours. After removal from the dye solution, the specimens were rinsed and dried. Then, roots were cut longitudinally following the principal axis of the root.

The roots were then photographed using a digital reflex camera (OLYMPUS EM 5). The distance to take pictures was standardized by a fixation device. The measurements were realized using ADOBE PS CS6 software. For more objectivity, many operators participated in the reading of the results. The Student's *t*-test was used to assess the results statistically and to compare differences in the depth of dye penetration between the three groups.

## 3. Results

In the negative control group, no sign of dye penetration was observed, which means there was no leakage.

In the positive control group, specimens showed maximum microleakage.

  Group 1: Revealed a dye leakage variable between 0 and 2.7 mm with an average of 1.0220 (±0.8467)
  Group 2: Revealed a dye leakage variable between 0 and 5 mm with an average of 1.3867 (±1.1344)
  Group 3: Revealed a dye leakage variable between 0.6 and 9.4 mm with an average of 2.2907 (±2.2781)

*P* > 0.05 means that there are no significant differences in the apical leakage between the three different apical preparation sizes (20, 30, and 50).

The average, variance, and standard deviation for the three experimental groups are demonstrated in Table 1.

## 4. Discussion

There are two topics where no consensus was established.

### 4.1. The Limit of the Apical Preparation

The three dimensional preparation of the root canal with no instrumental excess is a well know principal, but many opinions were established about the choice of the appropriate apical limit [14].

#### 4.1.1. The Apical Constriction

Apical constriction is a natural barrier which represents the smallest apical diameter and is located 0.5–1.5 mm inside the apical foramen [15]. This structure could coincide with the dentinocemental junction or very close to it. So, they are considered as a single anatomohistological entity elected as the apical limit of preparation [16, 17].

In fact, it would be appropriate to stop the shaping at the level where pulp tissue ends and leave the cement cone to permit a cemental reparation after the endodontic treatment. Therefore, the apical constriction (or CDJ) seems to be the ideal apical limit to be used as a matrix to support the closing material and avoid any overfilling [15–17].

#### 4.1.2. The Apical Foramen

The working length is initially established as close as possible to the canal exit or slightly short of the apical foramen to clean the entire length of the canal [18]. With this choice, we avoid to leave a noninstrumented apical area with bacteria and dentin shavings stemming from the preparation [19].

However, since the root apex can be subject to several morphological variations especially in the presence of apical resorption, files placed to the apical extent of the root as viewed radiographically will likely be outside the confines of the canal and create potential damage such as a persistent inflammation at apical area [18].

Both of these two positions provide no evidence-based data to support them as the ideal working-length technique for instrumentation and obturation [18]. A meta-analysis evaluation of success/failure showed a better success rate when the obturation was confined to the canal space [8].

While the guideline of 1-2 mm from the radiographic apex remains rational, the point of apical end of the preparation and obturation remains empirical [20]. The need to compact the gutta-percha and sealer against the apical dentin matrix (apical constriction) is essential for success. Also, to prevent extrusion, the cleaning and shaping procedures must be confined to the radicular space. Canals filled to the radiographic apex are actually overextended [20].

### 4.2. Degree of Apical Enlargement

Nowadays, two concepts oppose the modalities of apical enlargement.

#### 4.2.1. An Important Enlargement

This concept is based on the presumption that the canal debridement would be improved by increasing the apical diameter [21]. It is justified by the fact that microorganismes colonize the dentinal tubules in a depth of 200 to 300 μm where they remain inaccessible [22]. Indeed, Falk and Sedgley have demonstrated that larger apical preparation ensures a higher efficiency of the irrigation solution and reduces the bacterial growth at the apical level [23]. Brunson et al. maintain that larger apical preparation increases the volume of the irrigation solution at the apical level and it eliminates the tissue debris [24]. Fornari et al. and De-Deus et al. report that the apical enlargement reduces the noninstrumented areas in the root canal [25, 26].

On the other hand, the study by coldero et al. did not show any significant difference in intracanal bacterial reduction with or without apical enlargement preparation and concluded that it was not necessary to remove dentine in the apical part of the root canal when a suitable coronal taper is achieved to allow efficient irrigation of the root canal system [27].

Thus, apical enlargement remains controversial until today: no study has ever shown a direct relationship between apical enlargement and clinical success or endodontic treatment failure.

In fact, the apical enlargement presents many inconveniences.

#### 4.2.2. The Apical Debris Extrusion Risk

According to the results of the . study by Silva et al. that compared 4 systems of the canal preparation in a continuous rotation and reciprocating motions, apical debris extrusion occurred during large root apical preparations regardless of the file design and different kinematic motions (rotary or reciprocating) [28].

Extrusion of contaminated debris may be an important issue in the outcome of endodontic treatment. Besides bacterial extrusion due to mechanical and chemical aggression may cause flare-ups, and could also be responsible of post-operative pain and acute peri-apical lesions [29].

The root canal filling is technically more difficult to achieve. Few studies have been published comparing the apical diameter of preparation and apical sealing.

The study by Yared and Dagher evaluates the influence of apical enlargement on the sealing ability of vertical compaction and reports that the size 25 file group showed significantly less apical leakage than the size 40 file group [30]. Similarly, another study of Yared and Dagher showed that the canals instrumented to a size 40 file had significantly more overextensions than those instrumented to a size 25 file [31]. The study of Gomes-Filho et al. showed that greater dye leakage occurred after disruption and enlargement of the apical foramen [8]. Mente et al. evaluated the influence of the apical enlargement on sealing ability and observed a positive correlation between large apex diameter and greater leakage [32].

The results of our study are in accordance with all these studies since the most important values of the colorant infiltration have been observed in the group of larger apical diameter (F5) even if the comparison is statically not significant.

#### 4.2.3. A Minimal Enlargement

This concept is the base of the crown-down technique and implies the maintenance of a narrow foramen. It has been shown that

- a canal with an initial small apical diameter and maintained during the shaping is not less cleaned, provided that a sufficient conicity is achieved [27]. In large canals, it is not a matter of increasing the diameter of the foramen, but rather increasing the conicity of the canal;
- the necessary apical conicity to ensure the renewal of irrigation solution at the apical level, and thus disinfection, is at least 6% to 8% [33]. The advantage of the crown-down technique is to allow the cleaning of the canal as the instruments progress in the apical direction, which limits the extrusion in the periapex;
- a continuously tapering preparation from the access cavity to apical foramen facilitates efficient delivery of irrigant and creates apical resistance, which maintains the gutta-percha in the canal space and reduces the possibility of overfilling [34];
- in order to preserve apical foramen and to avoid the creation of apical zips or transport, it is important that instrumentation is performed according to all the principles of root canal therapy. Apical disruption violates one of these principles, which is to maintain the apical foramen in its original position [8].

## 5. Conclusion

Within the limitations of this study, there was no significant difference in the apical leakage between the three different apical preparation diameters. However, the most important infiltration was observed in the group prepared with the largest apical diameter.

There is still no real consensus to the exact size of the apical preparation. Moreover, it is necessary to construct a sufficient tapering along the root canal allowing the efficiency of the chemomechanical cleaning of the apical third and to guaranty a deep penetration of the irrigation, while preserving as much as possible the integrity of the noble anatomical structures of this apical area.

## Figures and Tables

**Table 1:** Calculation of the average, variance, and standard deviation of the dye penetration depths in the 3 experimental groups.

	Average (mm)	Variance	Standard deviation
Grp 1 (F1)	1.0220	0.7168	0.8467
Grp 2 (F3)	1.3867	1.2870	1.1344
Grp 3 (F5)	2.2907	5.1899	2.2781
		*P* value	0.08108
